# Medicinal Practice of Bioactive Compounds (Natural/Synthetic): An Insight into Gastrointestinal Disorders

**DOI:** 10.1155/2014/401698

**Published:** 2014-04-07

**Authors:** Mahmood Ameen Abdulla, Ibrahim Banat, Patrick Naughton

**Affiliations:** ^1^Department of Biomedical Sciences, Faculty of Medicine, University of Malaya, 50603 Kuala Lumpur, Malaysia; ^2^School of Biomedical Sciences, Faculty of Life and Health Sciences, University of Ulster, Coleraine BT52 1SA, UK

During the past two decades, the identification of new scientific developments to improve outcomes in gastrointestinal disorders has been attractive to many researchers. Pharmaceutical industries are now more motivated to introduce novel therapeutic remedies in the treatment of gastrointestinal disorders. Such disorders have increased at an exponential rate in various patient communities and both natural and synthetic compounds have been investigated for their potential biological activity in the treatment of these gastrointestinal disorders.

Several interesting works were assessed in this special issue. Amongst them five articles were chosen based on their critical findings in gastrointestinal disorders.

The effects of rikkunshito on the decrease in food intake were assessed after induction of stress in mice and showed improvement in the decrease of food intake probably via serotonin 2B receptor antagonism of isoliquiritigenin. In another work, the preventive effect of inositol hexaphosphate extract of rice bran on colon cancer was assessed. The results showed significant reduction in the expression of *β*-catenin and COX-2 in colon tumors. The Schiff base metal derivatives also may enhance the expression of HSP70 and suppress the expression of BAX proteins in an acute hemorrhagic gastric ulcer model. Another animal study assessed the hepatoprotective activity of the ethanolic extract of rhizomes of* Z. officinale* against thioacetamide-induced hepatotoxicity in rats. The floating dosage form of an anticancer drug was prepared in another study entitled “Preparation and Characterization of a Gastric Floating Dosage Form of Capecitabine.” The work characterized the sustained release tablet in terms of total floating time, dissolution, friability, hardness, drug content, and weight uniformity to compare the prepared formulation with the commercial tablet in terms of drug release, and to evaluate the stability of the formulation.

By presenting these articles, we hope that this issue incorporates new scientific evidence and emerging developments as the basis of rational treatment in medicinal practice using novel therapeutics (natural or synthetic compounds) in the treatment of gastrointestinal disorders.

*Mahmood Ameen Abdulla* *Ibrahim Banat* *Patrick Naughton*

